# Statistical methods for detecting and comparing periodic data and their application to the nycthemeral rhythm of bodily harm: A population based study

**DOI:** 10.1186/1740-3391-8-10

**Published:** 2010-11-08

**Authors:** Armin M Stroebel, Matthias Bergner, Udo Reulbach, Teresa Biermann, Teja W Groemer, Ingo Klein, Johannes Kornhuber

**Affiliations:** 1 Department of Psychiatry and Psychotherapy, University of Erlangen-Nuremberg, Schwabachanlage 6, 91054 Erlangen, Germany; 2 Department of Public Health and Primary Care, Trinity College Centre for Health Sciences, Adelaide and Meath Hospital, incorporating the National Children's Hospital, Tallaght, Dublin 24, Ireland; 3 Department of Statistics and Econometrics, University of Erlangen-Nuremberg, Lange Gasse 20, 90403 Nuremberg, Germany

## Abstract

**Background:**

Animals, including humans, exhibit a variety of biological rhythms. This article describes a method for the detection and simultaneous comparison of multiple nycthemeral rhythms.

**Methods:**

A statistical method for detecting periodic patterns in time-related data via harmonic regression is described. The method is particularly capable of detecting nycthemeral rhythms in medical data. Additionally a method for simultaneously comparing two or more periodic patterns is described, which derives from the analysis of variance (ANOVA). This method statistically confirms or rejects equality of periodic patterns. Mathematical descriptions of the detecting method and the comparing method are displayed.

**Results:**

Nycthemeral rhythms of incidents of bodily harm in Middle Franconia are analyzed in order to demonstrate both methods. Every day of the week showed a significant nycthemeral rhythm of bodily harm. These seven patterns of the week were compared to each other revealing only two different nycthemeral rhythms, one for Friday and Saturday and one for the other weekdays.

## Background

Analysis of biological activities that fluctuate throughout the day is common in various fields of medicine. Blood pressure and heart rate as well as the occurrence of acute cardiovascular disease are subject to a twenty-four hour rhythm (also referred to as circadian or nycthemeral rhythm) [1,2]. This rhythm is also present in episodes of dyspnoea in nocturnal asthma [3], intraocular pressure [4,5], and hormonal pulses [6-8]. Nycthemeral fluctuations in neurotransmitters and hormones have been discussed as influencing human behavior [9-11]. Suicide as well as parasuicide and violence against the person show day-night variation [12-14]. Assaults presenting to trauma centers display a distinct nycthemeral pattern [8-12]. In this study the nycthemeral rhythm of violent crime rates is analyzed to demonstrate a detection method and a comparison method suitable for twenty-four hour time series, but not limited to this sampling period.

Much mathematical effort was invested to detect and model the dependency on the time of day [15-19]. A classification of the data by identifying similarities and distinctions requires statistical methods [20-25].

The cosinor analysis is a common approach [26] that describes data by a single cosine function with fixed frequency plus a constant (single-harmonic model) yielding the three parameters amplitude, phase and mean [27]. Corresponding parameters were compared one by one to compare two or more time series modeled by cosinor analysis [28,29]. A multivariate technique is applied in this study aiming to compare several periodic patterns simultaneously. Models allowing more than one frequency (multi-harmonic model) show no graphic equivalent for the parameters amplitude and phase. Multi-harmonic models have been used to describe human core-temperature [18], blood pressure and incidence of angina [23] as well as in the nycthemeral distribution of violent crime rates, although the true waveform of nycthemeral rhythms is still a matter of debate. The purpose of this study is to identify the underlying frequencies and to compare the resulting periodic patterns via Fourier transform. This transform is common use in various fields of medicine [16] as well as other scientific areas. The explained variance of individual oscillations is utilized to detect the inherent periodic patterns of the data.

A modification of the analysis of variance (ANOVA) is used to compare two or more time series with periodic patterns. The typical ANOVA tests whether the means of several groups are equal. The scope of ANOVA is extended to periodic patterns by combining it with Fourier analysis. This new test rejects or confirms equality of multiple oscillating time series.

To demonstrate both methods, the oscillations of violent crimes in Middle Franconia, Bavaria/Germany from 2002 to 2005, were analyzed. Nycthemeral rhythms of bodily harm were identified on all seven days of the week. The seven patterns of the week were compared to each other revealing only two different nycthemeral rhythms. We demonstrate that the nycthemeral rhythms on Friday and Saturday are equal and differ significantly from the rhythms of the other weekdays, which are then equal again.

To compare our method with the cosinor method an analysis of the same data is performed and yields no strong evidence of different rhythms.

The simultaneous comparison of a greater number of nycthemeral rhythms is made possible by the use of the mathematical methods described in this study. A need for such procedures derives from the prospect of developing a prediction model for violent crime rates which is of immediate interest for public services such as social facilities, police departments and hospitals.

The section detection method contains a procedure to find the inherent frequencies of the data, the section Fourier Anova describes the comparison method, the results section illustrates both methods by analyzing nycthemeral rhythms of offenses against the person causing bodily harm and in the conclusion limitations, modifications and alternatives to our methods are discussed.

## Methods

### Detection method

A statistical test for finding the frequencies of oscillating data is described. Using harmonic frequencies the data are modeled as a sum of sine and cosine oscillations and a Fourier transform is performed. In our case the Fourier transform equals an ordinary least squares. All frequencies are tested for significance. The ratio of explained variance of a frequency and remaining variance acts as test statistic. Model selection is carried out by a Bonferroni-Holm Method (see [30]).

Fitting harmonic models to nycthemeral rhythms is a common procedure [31-33]. The detection method is ancillary, its output is used as input for the comparison method (see section Fourier ANOVA). From a numerical vantage point linear least squares with orthonormal regressors are applied. From a linear algebra perspective we choose a specific set of vectors forming an orthonormal basis and change basis. Statistical methods are applied to search for single coordinates of the data (relative to the new basis) that are „large“ compared to the other coordinates. The orthonormal basis ensures independent and normal distributed regression coefficients; thus choosing significant frequencies (i.e. model selection) is straightforward. Furthermore the orthonormal regressors are necessary for our extension of ANOVA described in the section Fourier ANOVA.

The model for our data is

(1) $$x_{t}=\sum_{j}a_{j}\cos(2\pi f_{j}t)+b_{j}\sin(2\pi f_{j}t)+\epsilon_{t}\;t=1\ldots n,$$

with white noise ϵ. Constant terms are omitted. So a time series sampled *n *times with a fixed sampling interval, homoscedasticity and uncorrelated noise and without a linear trend or missing values is assumed. The regressors have the harmonic frequencies

(2) $$f_{j}=\frac{j}{n}\text{,}\;\;j=1\ldots \left\lfloor \frac{n}{2}\right\rfloor .$$

By this choice the regressors cos(2*πf_j_t*) and sin(2*πf_j_t*) are an orthogonal basis of *R^n^*. Estimating *a *and *b *with ordinary least squares against the normalized regressors yields independent and normal distributed coefficients. To determine significant frequencies we search for large coefficients *a *and *b *by a method similar to a Wald-statistic and by a Bonferroni-Holm procedure [30].

The null hypotheses are $H_{0}^{j}$: *a_j _*= *b_j _*= 0, or: „no significant periodic pattern with frequency *f_j _*in the data“. To test these hypotheses

(3) $$c_{j}^{2}=a_{j}^{2}+b_{j}^{2}$$

is calculated, mimicking a periodogram. The value $c_{j}^{2}$ can be interpreted as the explained variance of frequency *f_j_*, furthermore *c_j _*is invariant under time-shift of the data. Then *c *is sorted in descending order a *F *-distributed test statistic is calculated:

(4) $$T_{j}=\frac{c_{j}^{2}}{\sum_{i<j}c_{i}^{2}}\sim F_{2,n-2j},\;j=1\ldots n-1$$

which is tested on the corrected significance level $1-{\left(1-\alpha \right)}^{\frac{1}{n-j}}\approx \frac{\alpha }{n-j}$. If *T_j _*does not exceed the critical value for a specific *j*, then all *T_i _*with *i > j *are not tested anymore. This test yields a set of significant frequencies. A Fourier approximation ℱ*_F _*of the data is obtained by evaluating equation 1 using only a subset *F *of the harmonic frequencies (e.g. the significant frequencies) and their corresponding amplitudes:

(5) $${ℱ}_{F}(x)=\sum_{f\in F}a_{f}\cos(2\pi ft)+b_{f}\sin(2\pi ft),\;t=1\ldots n.$$

The Fourier approximation filters the periodic components out of the data; it is a denoising procedure. The data is decomposed in a fundamental frequency and its multiple, the harmonics. The Fourier coefficients indicate the strength, i.e. the amplitude of these oscillations. Usually the fundamental frequency has the highest amplitude and the strength decreases for greater harmonics. The influence of the harmonics can reach from only small adjustments of the fundamental oscillations to generating additional maxima, minima or plateaus.

### Comparison method (Fourier ANOVA)

A statistical test for comparing periodic patterns of grouped data is described. The test determines if the rhythm of the groups are equal or not. The mathematical concept of the ANOVA is transferred to periodic patterns by substituting the mean estimators for Fourier approximations. This test compares the periodic patterns in its entirety. The orthogonal regressors mentioned in the section Detection method are necessary for this test.

Suppose data divided in *k *groups with *n *measurements for every group and denote this data as *x_t,j _*(*t *= 1 ... *n, j *= 1 ... *k*). The *F *distributed ANOVA test statistic for equal means in every group is

(6) $$\frac{\frac{1}{df_{1}}\sum_{t,j}{\left(\bar{x_{.,j}}-\bar{x_{.,.}}\right)}^{2}}{\frac{1}{df_{2}}\sum_{t,j}{\left(x_{t,j}-\bar{x_{.,j}}\right)}^{2}}.$$

To compare not the means but the periodic pattern of every group we substitute the mean estimators for the Fourier approximation (see 5):

(7) $$T_{F}(x)=\frac{\frac{1}{df_{1}}\sum_{t,j}{\left({ℱ}_{F}(x_{.,j})-{ℱ}_{F}(x_{.,.})\right)}^{2}}{\frac{1}{df_{2}}\sum_{t,j}{(x_{t,j}-{ℱ}_{F}(x_{.,j}))}^{2}}\sim F_{df_{1},df_{2}}.$$

The frequencies *F *are chosen as described in the section Detection method: the detection method is applied to every group of *x*. Testing with *d *= |*F*| frequencies the degrees of freedom are *df*_1 _= 2*dk *- 2*d *and *df*_2 _= *nk *- 2*dk*.

The test uses the same idea as the ANOVA: Calculate the variance within the groups, i.e. the deviation of the data from its Fourier approximation within every group. Furthermore calculate the variance between the groups, i.e. the deviation the Fourier approximation of the single groups and the Fourier approximation of the whole data. If all groups show the same rhythm then the variance between the groups should have roughly the same magnitude as the variance within the groups. Conversely a large variance between the groups argues for an impact of a group on the rhythm.

In the following we will scrutinize the distribution of the test statistic in equation 7: We show that the test statistic *T_F _*in equation is *F *distributed. Cochran's Theorem, as stated in [34], yields a *χ*^2 ^distribution of the nominator and the denominator of equation 7. To apply this theroem the test statistic needs a matrix representation.

The Fourier approximation in equation 5 has a matrix representation: For *f *∈ ℝ define the column vectors

(8) $$\begin{array}{l} c_{f}^{n}\;:=\;k_{c}(f,n)\;{\left(\cos(2\pi ft)\right)}_{t=0\ldots n-1}\\ s_{f}^{n}\;:=\;k_{s}(f,n)\;{\left(\sin(2\pi ft)\right)}_{t=0\ldots n-1} \end{array}$$

with normalization constants *k_c_*(*f, n*)*, k_s_*(*f, n*). Then then Fourier approximation can be written as

(9) $${ℱ}_{F}(x)=\left(\sum_{f\in F}s_{f}^{n}{\left(s_{f}^{n}\right)}^{T}+c_{f}^{n}{\left(c_{f}^{n}\right)}^{T}\right)x.$$

Let $M_{F}^{n}$ be this transformation matrix of ℱ*_F_*, then $M_{F}^{n}$ is a symmetric projection, i.e.

(10) $${\left(M_{F}^{n}\right)}^{2}=M_{F}^{n},\;\;{\left(M_{F}^{n}\right)}^{T}=M_{F}^{n}.$$

Furthermore pile the columns of the data *x *∈ *R*^*n,k *^one below the other and call this vector *y ∈ R^nk^*. Define the matrices

(11) $$A_{1}:=M_{F}^{nk}\in {\mathbb{R}}^{\left(nk)\times (nk\right)}$$

and

(12) $$A_{2}\text{:}=\left(\begin{array}{cccc} M_{F}^{n} & & & 0\\ & M_{F}^{n} & & \\ & & \ddots & \\ 0 & & & M_{F}^{n} \end{array}\right)\in {\mathbb{R}}^{\left(nk)\times (nk\right)}.$$

Because *A*_1 _and *A*_2 _are symmetric projections the test statistic *T_F _*in equation 7 can be written as

(13) $$\begin{array}{c} T_{F}(x)=\frac{\frac{1}{df_{1}}|(A_{2}-A_{1})y{|}^{2}}{\frac{1}{df_{2}}|(1-A_{2})y{|}^{2}}\\ =\frac{\frac{1}{df_{1}}\langle (A_{2}-A_{1})y,(A_{2}-A_{1})y\rangle }{\frac{1}{df_{2}}\langle (1-A_{2})y,(1-A_{2})y\rangle }\\ =\frac{\frac{1}{df_{1}}y^{T}{\left(A_{2}-A_{1}\right)}^{T}(A_{2}-A_{1})y}{\frac{1}{df_{2}}y^{T}{\left(1-A_{2}\right)}^{T}(1-A_{2})y}\\ =\frac{\frac{1}{df_{1}}y^{T}(A_{2}-A_{1})y}{\frac{1}{df_{2}}y^{T}(1-A_{2})y}. \end{array}$$

Now the test statistic has a representation suitable for Cochran's Theorem. All that is left is the orthogonality assumption for the projections *A*_2 _- *A*_1 _and $1-A_{2}$. The specific form of the harmonic frequencies is again utilized: The image of *A*_1 _is spanned by $s_{f}^{nk}$ and $c_{f}^{nk}$ (*f *∈ *F *). The image of *A*_2 _is spanned by the vectors $s_{f(j)}^{n}$ and $c_{f(j)}^{n}$ filled up with the zero vector **0 **= (0...0) ∈ ℝ*^n^*:

(14) $$\begin{array}{l} (\underset{m\text{ times}}{\underset{︸}{0\ldots 0}}c_{f(j)}^{n}\underset{k-1-m\text{ times}}{\underset{︸}{0\ldots 0}})\in {\mathbb{R}}^{nk},\;f\in F,\;m=0\ldots k-1,\\ (\underset{m\text{ times}}{\underset{︸}{0\ldots 0}}s_{f(j)}^{n}\underset{k-1-m\text{ times}}{\underset{︸}{0\ldots 0}})\in {\mathbb{R}}^{nk},\;f\in F,\;m=0\ldots k-1. \end{array}$$

By definition of the harmonic frequencies (see equation 2) the following equation holds except for normalization factor:

(15) $$\begin{array}{l} s_{f}^{nk}=(\underset{k\text{ times}}{\underset{︸}{s_{f}^{n},\ldots ,s_{f}^{n}}})\\ c_{f}^{nk}=(\underset{k\text{ times}}{\underset{︸}{c_{f}^{n},\ldots ,c_{f}^{n}}}). \end{array}$$

So the image of *A*_1 _is a subset of the image of *A*_2 _and it holds:

(16) $$A_{1}A_{2}=A_{2}A_{1}=A_{1}.$$

This equation shows the orthogonality of the projections of Cochran's Theorem.

## Results

Nycthemeral rhythm of violent crime rates are analyzed to demonstrate both the detection and comparison method.

The study included 15881 crimes of violent behavior (without suicides) which were filed at the Police Department of Middle Franconia, Bavaria/Germany between January 1, 2002 and December 31, 2005, and gathered into the EVioS (Erlangener Violence Studies [35]) data base. Bodily harm as defined in § 223 German Criminal Code is more closely examined. We investigate if the seven days of the week show different nycthemeral rhythms of bodily harm. Data handling and calculations were performed by Microsoft Excel^®^, Matlab^® ^and R. Significance level was set to 0.05.

**Figure 1:**
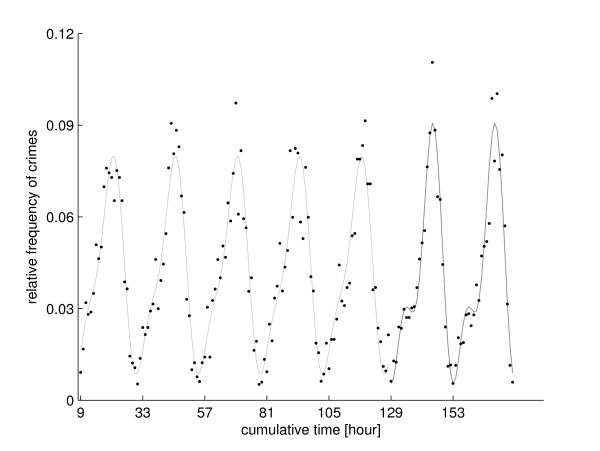
**Normalized crime rates and its Fourier approximations**. Black dots show the relative frequency of 11402 crimes of bodily harm committed in the years 2002 to 2005 in Middle Franconia, Bavaria/Germany during the 168 hours of a week, starting Sunday at 9:00 a.m. Nycthemeral rhythms are visible. Solid line show the Fourier approximation of relative number of crimes versus cumulative time in hours, starting at 9:00 a.m. Light gray line shows the Fourier approximations of normalized crime rates for *d*1 to *d*5 (Sunday 9:00 a.m. to Friday 9:00 a.m.); the dark gray line for *d*6 and *d*7 (Friday 9:00 a.m. to Sunday 9:00 a.m.). A difference of these two rhythms is a shift of the maxima from 10:00 p.m. to 1:00 a.m. Furthermore the maxima of the second rhythm are higher than those of the first.

In the following, the detection method shows the existence of nycthemeral rhythms of bodily harm on all seven days of the week. A comparison of these seven rhythms reveals only two different nycthemeral rhythms, one describing crime rates on Friday and Saturday, the other on Sunday to Thursday. In order to analyze a more homogeneous sample, only crimes committed by male offenders and not occurring on holidays such as New Year's Eve are further surveyed; this sample consists of 11402 cases. The investigated data *x *∈ ℝ^24 × 7 ^are the number of violent acts *x*(*h, d*) at a specific hour *h ∈ *{1 ... 24} and „day“ *d *∈ {1 ... 7}. We define the first „day“ as the 24 hours starting Sunday at 9:00 a.m. and denote it with *d*1. This definition is adapted to the data: at 9:00 a.m. violent crime rates of all seven days are similar and a renewal of the time series occurs (see Figure 1). Furthermore the second „day“ *d*2 is defined as the 24 hours starting Monday at 9:00 a.m. lasting till Tuesday 9:00 a.m. and so forth.

**Figure 2:**
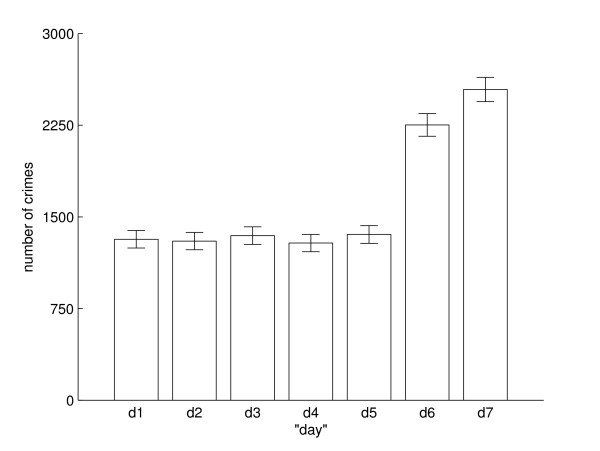
**Distribution of crimes of bodily harm on the seven days of a week**. Distribution of the 11402 crimes of bodily harm committed in the years 2002 to 2005 in Middle Franconia, Bavaria/Germany on the seven „days“ of a week, with 95% confidence intervals. *d*1 is the 24 hour timespan starting at Sunday 9:00 a.m. and ending at Monday 9:00 a.m. and so on.

The histogram in Figure 2 shows the distribution of violent crimes per „day“ with 95% confidence intervals. In particular the number of crimes on *d*6 and *d*7 are distinct. We are interested in the nycthemeral rhythm and not in total numbers; so we normalize the data by dividing the number of crimes at „day“ *d *and hour *h *by the number of crimes on „day“ *d*. The normalized data are called *y *∈ ℝ^24 × 7^. So every column of *y *sums up to 1 and thus can be interpeted as relative frequency of crimes.

The assumptions of our model in equation 1 are satisfied by the data *y*: There is no trend or missing values and a constant time between two consecutive samples. *x *consists of count data, so *x*(*h, d*) follows a Poisson distribution and the normalized data *y*(*h, d*) are well approximated by a normal distribution. The sequence *y*(*h*, *d*)_*h *= 1 ... 24,*d *= 1 ... 7 _is assumed to be independent, because sites of crimes are spatially separated or offenders don't even know each other. Homoscedasticity (constant variance of the residuals) and Poisson distributions do not make a good match: For Poisson random variable the mean equals the variance and we assume a oscillating number of crimes. So the residuals will not automatically be homoscedastic and are afterwards tested for „whiteness“ by a Kolmogorov-Smirnov test [36], a Lilliefors test [37] (both for normal distribution), a Breusch-Godfrey test [38,39] and a Wald Wolvowitz runs test [40] (for absence of autocorrelation, the latter is applied to the signs of the residuals). The data are also tested for stationary cycles by a Canova-Hansen [41] test and a Kwiatkowski-Phillips-Schmidt-Shin test [42]. We also divided the data in 10 disjoint random subsamples to avoid testing hypotheses suggested by the data.

**Figure 3:**
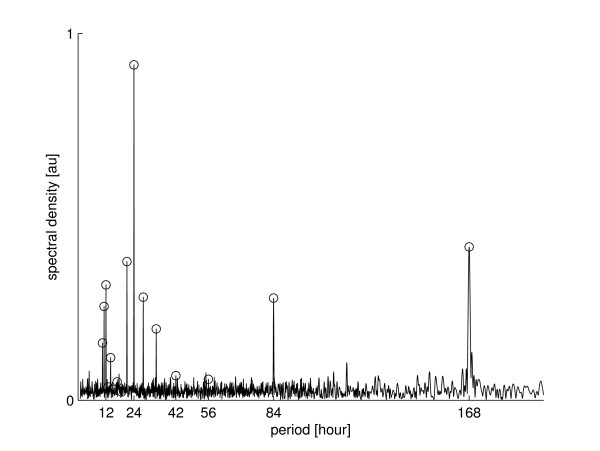
**Periodogram of incidents per hour**. The periodogram is applied to the 35064 hours of the four year sampling period. The period of 168 hours (one week), 24 hours (one day) and their corresponding harmonic frequencies are tagged with circles. All high peaks of the spectral density coincide with these frequencies. The other shown periods have relatively small density.

Applying the detection method to the columns of *y *reveals significant nycthemeral rhythm on every „day“. All seven „days“ showed significant periods of length 24 and 12 hours except *d*3 and *d*4, which showed only a significant 24 hour period. So every „day“ shows a nycthemeral rhythm of bodily harm. Note that by analyzing single days of the week, i.e. columns of *y*, which have a length of 24, we restrict our search to the frequency $\frac{1}{24h}$ and its integer multiple (see the model in equation 1 and its description). We have two reason for doing so: first we have a priori knowledge: The sun is a zeitgeber for the human biological clock [43], that argues for a 24 hour rhythm. Furthermore the week is the time unit that governs the working life in Germany and separates it in five working days (Monday to Friday) and two weekend days (Saturday and Sunday). Second we get a posteriori knowledge: by applying our detection method to the whole data *y *which revealed no other significant periods, especially no significant period greater than 24 hours and by calculating a periodogram of the data (see Figure 3), which reveals only a day period, a week period and their corresponding harmonics.

Applying the comparison method to *d*1 to *d*7 (frequencies $f=(\frac{1}{24},\;\frac{1}{12})\in {\mathbb{R}}^{2}$ and number of samples *n *= 7·24) generates a p-value smaller than 0.05 (*F *= 18.1639, *df*_1 _= 24, *df*_2 _= 140). So there are at least two different periodic patterns in the data. This finding is verified in the 10 randomly-generated subsamples: comparing the period of the subsamples yields p-values within the interval [1.04 · 10^-10^, 1.3 · 10^-3^].

Comparing *d*6 and *d*7 (*n *= 2 · 24, *f *as above) yields a p-value of 0.3582 (*F *= 1.1352, *df*_1 _= 4, *df*_2 _= 40). So there is no significant difference between *d*6 and *d*7. So Friday and Saturday show the same nycthemeral rhythm of bodily harm. By testing this hypothesis in the 10 subsamples p-values in the interval [0.15, 0.98] are obtained.

We found that nycthemeral rhythm of *d*6 and *d*7 is different from the rhythm of *d*1 to *d*5. For statistical verification, the comparison method was applied to the 93 partitions *P *⊂ {1...7} of the seven days, that contain at least one element of {1, 2, 3, 4, 5} and at least one element of {6, 7}. So we tested the 93 hypothesis *H_P _*: „There is no significant difference between the „days“ of partition *P*“. The comparison method yields p-values smaller than 8.5 · 10^-11^. Bonferroni's inequality yields an upper bound for the p-value of the hypothesis ∪ *H_P _*(„There is at least one of the 93 partitions without significant difference between the nycthemeral rhythms of the „days“ of this partition“): *P *(∪*H_P _*) < 93 · 8.5 · 10^-11 ^*<*0.05 and thus reject this hypothesis. We accept the alternative hypothesis: „the „days“ of all 93 partitions have significant different nycthemeral rhythms“.

Comparing only *d*1 to *d*5 (*n *= 5 · 24, *f *as above) yields a p-value of 0.0515 (*F *= 1.7372, *df*_1 _= 16, *df*_2 _= 100). Applying this test to the 10 subsamples yields p-values within [0.0457, 0.93], one p-value was lower than 5%. Testing the 26 partitions of {1 ... 5}, which have at least two elements yields p-values ranged from 0.0047 to 0.9908, none was smaller than Bonferroni-corrected significance level $\frac{5\%}{26}=0.0019$. Altogether we found some significant differences within *d*1 to *d*5, but consider them marginal. So there are only two significantly different nycthemeral rhythms, one describing crime rates on *d*6 and *d*7, the other on *d*1 to *d*5, see Figure 1 for a plot of these two rhythms.

**Figure 4:**
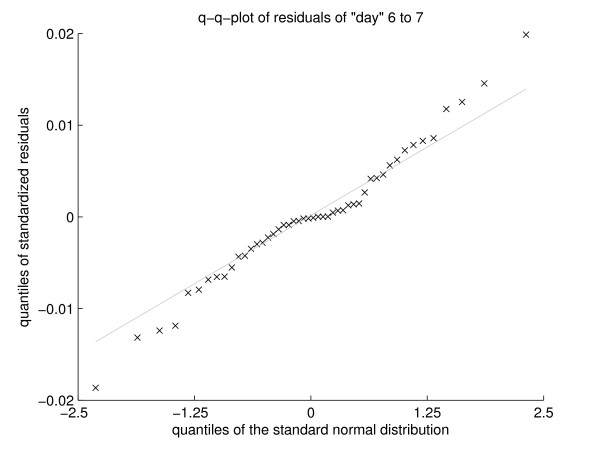
**Quantil-quantil-plot of residuals**. Quantil-quantil-plot of residuals of *d*6 and *d*7 against standard normal quantiles (black cross). The gray line joins the first and the third quartile. The absence of large deviations between the black crosses and the gray line implies a normal distribution of the residuals.

Now the „whiteness“ of the residuals of the fit of *d*1 to *d*5 is tested. Figure 4 shows a quantile-quantile-plot of the residuals against a standard normal distribution, which is almost linear, arguing for normal distributed residuals. A formal test for normal distribution is the Kolmogorov-Smirnov test. Testing the residuals divided by their estimated standard deviation against the standard normal distribution yields *p *= 0.96, *d_ks _*= 0.0440, *n *= 120.

Autocorrelation of the residuals biases the estimation of the coefficients and is a evidence for a misspecified model. A Breusch-Godfrey test for autocorrelation up to order 23 does also not reject the null hypothesis (*p *= 0.155, $\chi_{23}^{2}$ = 29.8). So these residuals show no significant autocorrelation.

Stationarity is a property often desired in time series analysis, particular in econometrics [44,45]. A stationary process fluctuates steadily around a deterministic trend, a nonstationary series is subject to persistent random shocks or can even be transient. If the variables in the regression model are not stationary, then the standard assumptions for asymptotic analysis may not be valid. In other words, the usual F-ratios will not follow a F-distribution, so we cannot validly undertake hypothesis tests about the regression parameters. The Canova-Hansen Test and the Kwiatkowski-Phillips-Schmidt-Shin Test did not reject the null Hypothesis of stationary seasonal cycles. Applying these tests to the residuals of the fit of *d*6 and *d*7 yields the same results (*p *= 0.369, *d_ks _*= 0.1290, *n *= 48 and *p *= 0.350, $\chi_{23}^{2}$ = 25.0, no rejection of the null Hypothesis by Canova-Hansen Test and Kwiatkowski-Phillips-Schmidt-Shin Test).

Though our Fourier approximation underestimates the peaked crime rates around midnight the coefficient of determination of the single days is within [0.86, 0.96]. Overall the model is satisfying.

## Conclusion

Two statistical methods that will enlarge the scientists toolbox for analyzing multi-harmonic oscillations were described. As the example demonstrated the methods can be used to detect and compare multi-harmonic patterns in biological rhythm data.

The orthogonality of the sine and cosine vectors is intensively used to calculate the exact distribution of certain test statistics, not just the approximate distribution for large sample sizes. But this orthogonality also limits the set of frequencies in our multi-harmonic model. In this special case our detection method is an extension of the cosinor-method to multi harmonic models. It also includes a model selection process. Our comparison method uses the whole periodic patterns instead of single parameters. This is an enhancement of the commonly used ANOVA with single parameter „mean“. Furthermore the exact distribution of the test statistic is known, not just an approximate or a limiting distribution for large sample sizes. This can in some cases increase the tests power. In addition the method allows a simultaneous comparison of several time series. This allows to test the hypothesis if „at least one time series shows a different rhythm“ without having any a priori knowledge which one could be deviant (this situation can occur if for example the study design or the data does not allow a partition in a control group and a treatment group).

Problems may occur with missing values (no ON-basis), trends in the data (model is not valid) or the choice of the number of samples, when no a priori knowledge of the inherent periods of the data is available. To derive a more robust version of the statistical test use the rank of the residuals instead the residuals analogous to the ANOVA on ranks. Identifying the method's limitations will help improve it and make it more universal, which is one of the reasons for providing a detailed description of the method calculation steps.

Likelihood ratio tests are in common use for model selection or hypothesis testing and could be an alternative to our tests. Least squares estimates of the coefficients coincide with the maximum likelihood estimates, if the residuals are normal distributed and homoscedastic. Our tests confirm, that the residuals have these properties. So there is neither a gain nor a loss in switching to likelihood ratio tests, which are based on maximum likelihood estimates. Furthermore only the limiting distribution of the likelihood ratio test statistic for large sample sizes is known, whereas the exact distribution of our test statistics is specified. The described detection method uses all harmonic frequencies, because potentially all frequencies could be inherent in the data. However this approach can increase the false negative rate of the test, because the corrected significance level becomes too small. So we are using a conservative test. As Albert and Hunsberger [31] point out there is a „wide range of circadian patterns which can be characterized with a few harmonics“ and that they „recommend choosing between one, two, or three harmonics“. We too found only two significant harmonics in our analysis and observed a good coefficient of determination and white noise residuals. So if some frequencies are ruled out by a priori knowledge the detection method can be executed with fewer harmonics to increase the tests power.

We compared our methods with the cosinor method [26], which fits a single cosine wave with a user defined period to the data: coefficient of determination is 0.732 for a 24 hour period and 0.2 for a 12 hour period when fitting Friday and Saturday. Our detection method achieved a coefficient of determination of 0.86. The cosinor method also calculated the amplitude of the 24 hour periods for workdays and weekends: they differed by only 5%. Analyzing the amplitudes of the first harmonic yields overlapping confidence intervals. So the cosinor method gives no strong evidence for different rhythms on workdays and weekends. A significant difference between workdays and weekends is revealed by simultaneously comparing all weekdays as we did in section.

The findings of a 24 hour period on every day could be for example associated with the hormones testosteron and serotonin. Both of them show a nycthemeral rhythm [7,8] and are linked to violent behavior [46,47]. The different rhythm on Friday and Saturday could be caused by exogenous factors like increased alcohol consumption [48].

## Competing interests

The authors declare that they have no competing interests.

## Authors' contributions

AS contributed to the conception and the design of the study, analyzed the data and drafted the manuscript. UR contributed to the conception and the design of the study. TB acquired the data. IK contributed to the analysis. JK contributed to the intellectual content. TG, MB and all other authors read and approved the final version of the article.
